# The effectiveness of the Moms’ Empowerment Program (MEP) in reducing post-traumatic stress symptoms and depressive symptoms among Iranian mothers surviving intimate partner violence

**DOI:** 10.1186/s40359-025-03819-1

**Published:** 2025-12-13

**Authors:** Omid Isanejad, Shirin NaghibAlsadate, Sandra A. Graham-Bermann

**Affiliations:** 1 Department of Counseling, University of Kurdistan, University of Kurdistan, Pasdaran Blvd, Sanandaj, 6617715175 Iran; 2 Professor of Psychology and Psychiatry, University of Michigan, Ann Arbor, MI 48109-1043 USA

**Keywords:** Moms’ empowerment program, Intimate partner violence, Post-traumatic stress, Depressive symptoms

## Abstract

**Purpose:**

Intimate partner violence (IPV) is a global health crisis with severe psychological sequelae. While the Moms’ Empowerment Program (MEP) is an evidence-based intervention, its efficacy in non-Western contexts remains under-explored. This study evaluated the effectiveness of a systematically, culturally adapted MEP in reducing post-traumatic stress symptoms (PTSS) and depressive symptoms among Iranian mothers who have survived IPV.

**Methods:**

A single-case experimental design (SCED) with an A-B-Follow-up framework was replicated across eight female participants (*N* = 8) recruited from community shelters. The intervention consisted of a 10-week, group-based MEP. PTSS and depressive symptoms were measured repeatedly across baseline, intervention, and a three-month follow-up phase using the PTSD Checklist for DSM-5 (PCL-5) and the Beck Depression Inventory-Short Form (BDI-SF).

**Results:**

Visual analysis revealed an immediate, marked, and consistent reduction in both PTSS and depression scores for all eight participants following the introduction of the intervention. Therapeutic gains were durably maintained throughout the follow-up period. The effects were of a large magnitude, with individual-level Cohen's d effect sizes ranging from 1.64 to 1.83.

**Conclusions:**

This study provides strong preliminary evidence that a culturally adapted MEP can be a highly effective intervention for treating the psychological wounds of IPV in a non-Western, Middle Eastern context. The findings underscore the critical importance of culturally sensitive, evidence-based care and support the program's potential for broader global application.

## Introduction

Intimate Partner Violence (IPV) stands as a pervasive global public health crisis and a profound violation of human rights, exacting severe human, social, and economic costs [1–4]. Defined by the Centers for Disease Control and Prevention (CDC) as physical or sexual violence, stalking, or psychological aggression by a current or former partner, IPV is a threat to the well-being of millions [5]. Globally, over a quarter (27%) of women aged 15–49 have endured physical or sexual violence from an intimate partner, a figure that underscores the scale of the epidemic [6]. While prevalence varies, the burden is particularly acute in certain regions. In Iran, for instance, the past-year prevalence of IPV among married women has been reported as high as 78.1%, highlighting a critical and urgent need for effective, context-specific interventions [7, 8].

The consequences of IPV are devastating, encompassing both acute physical injuries—such as fractures and traumatic brain injury—and a constellation of chronic psychological disorders [9, 10]. Among the most debilitating of these sequelae are depression and post-traumatic stress symptoms (PTSS), which disproportionately affect female survivors [11, 12]. The link between IPV and maternal mental health is particularly pernicious. Depression, characterized by a persistent loss of interest or pleasure [13], is not only a leading cause of disability in women worldwide [14] but is also significantly exacerbated by the experience of violence [15, 16]. Concurrently, the chronic stress and terror inherent in abusive relationships often manifest as PTSD, a condition marked by intrusive memories, avoidance, and hyperarousal that severely impairs daily functioning [13, 17, 18]. The convergence of these disorders in mothers who have experienced IPV creates a compound crisis, impacting not only the woman's own health but also her capacity to parent, thereby risking the intergenerational transmission of trauma. This intergenerational transmission of trauma underscores the importance of interventions that not only target clinical symptoms but also enhance the mother's overall sense of personal wellbeing, which is strongly linked to recovery from PTSD [19].

In response to this crisis, a range of therapeutic interventions have emerged since the 1970 s, many of which have successfully reduced depressive symptoms and PTSS in survivors [20, 21]. Early efforts, such as those by Sullivan and colleagues, established the value of social support and resource access in improving survivors' quality of life and self-esteem [22–24]. Furthermore, contemporary approaches have begun to explore the integration of technology to enhance the reach and flexibility of interventions for vulnerable populations, such as using video-based support during perinatal home visitation [25]. Building on this foundation, more structured, psychoeducational group interventions have been developed. Among the most promising is the Moms’ Empowerment Program (MEP), a manualized, 10-session, strengths-based group intervention grounded in Interpersonal Psychotherapy (IPT) [26]. The MEP is specifically designed to enhance mothers' coping mechanisms, reduce stress, and directly target symptoms of depression and PTSS. Randomized controlled trials in Western contexts have demonstrated its efficacy in improving both maternal mental health and parenting practices [20, 27].

The core idea of this intervention is its strategic emphasis on the parental role—not only as a collection of behaviors, but as a significant and intrinsic component of a mother's identity, frequently impacted by intimate partner abuse. The psychological consequences of intimate partner violence (IPV) primarily undermine a mother's self-efficacy and personal agency, as elucidated by the learned helplessness hypothesis [28]. The MEP utilizes the parental function as the fundamental basis for reconstructing this agency. This highlights the significant importance of self-efficacy theory [29], which asserts that mastery experiences are the most powerful catalysts for psychological transformation. The curriculum systematically offers mothers a variety of tangible parenting techniques to facilitate mastery experiences. The subsequent restoration of self-efficacy in parenting is believed to initiate a positive feedback loop, influencing other aspects of life and fostering an enhanced sense of overall competence [26]. This process is enhanced by the reduction of secondary stressors, especially the difficult kid behaviors that are often intensified in the tumultuous environments of violent households [30]. Mitigating this everyday stress burden directly addresses the hyperarousal and emotional dysregulation characteristic of PTSS [19]. Thus, improving parental competence is viewed not merely as a supplementary advantage of the intervention, but as the primary catalyst for therapeutic progress—a direct route to reinstating agency, alleviating everyday suffering, and eventually, reducing the deep-seated effects of trauma.

Despite the demonstrated success of the MEP in North America, a significant gap remains in the literature regarding its applicability and effectiveness in non-Western, culturally distinct contexts. Beyond the high prevalence rates, ranging from 30 to 90% [31], the experience of domestic violence and subsequent help-seeking behaviors in the Iranian context are shaped by a unique constellation of socio-cultural factors. These include a strong cultural emphasis on preserving family privacy and cohesion, which can create significant barriers to disclosing abuse outside the family unit [32]. Furthermore, the social stigma associated with divorce or the label of victimhood can lead to profound social isolation and deter women from seeking formal support systems [7]. Consequently, help-seeking pathways often diverge from Western models, with many women initially turning to informal support networks such as family and trusted community elders, while formal mental health services may be perceived as less accessible or culturally incongruent [8]. It is within this complex milieu that a Western-developed intervention like the MEP cannot be merely translated and implemented verbatim. Its efficacy is contingent upon a deep cultural adaptation that respects local values, directly addresses barriers like isolation by fostering a safe group environment, and frames concepts such as empowerment in a manner that resonates with the lived realities of Iranian mothers. Therefore, the present study sought to address this critical gap by implementing and evaluating a culturally adapted version of the Moms’ Empowerment Program (MEP) for a sample of Iranian mothers who have survived intimate partner violence. The primary objective was to determine the program's efficacy in reducing symptoms of depression and post-traumatic stress. We hypothesized that participants in the MEP would show a statistically significant reduction in both depression and PTSS scores from pre-intervention to post-intervention and follow-up.

## Method

### Research design

This study employed a quasi-experimental, single-case experimental design (SCED) to evaluate the intervention's efficacy. Specifically, we utilized an A-B-Follow-up design replicated across eight participants. This framework involves a baseline phase (A) with repeated pre-intervention measurements, an intervention phase (B) during which the treatment was administered, and a subsequent follow-up phase to assess the maintenance of effects. By demonstrating a consistent and marked change in the level or trend of the dependent variables immediately following the introduction of the intervention across multiple, independent replications, this design allows for an inference of a functional relationship between the treatment and the observed outcomes. The independent variable was the culturally adapted Moms’ Empowerment Program (MEP), and the dependent variables were PTSS and depressive symptoms.

#### Participants

The study sample comprised eight mothers who had survived IPV. Participants were recruited through a purposive sampling strategy in collaboration with administrators at two local women's shelters in Sanandaj, Iran. The recruitment process followed a multi-step, ethically sensitive protocol. Initially, shelter administrators, with prior blanket consent from residents for internal program reviews, conducted a preliminary, anonymized review of case files to identify potentially eligible individuals based on broad criteria (e.g., being a mother, history of IPV). Following this, a trained female member of the shelter staff, who had an established rapport with the residents, approached 12 potentially eligible women privately to introduce the study and inquire if they would be willing to speak with a member of the research team.

Ten women agreed to a confidential, introductory meeting with the second author (a trained female researcher), during which the study's objectives, procedures, voluntary nature, and stringent confidentiality measures were thoroughly explained. Of these, eight women met the full inclusion criteria and provided written informed consent to participate. All eight participants completed the entire study protocol, resulting in no attrition. The final sample had a mean age of 34.50 years (SD = 8.63). The majority (75%) were divorced and reported low monthly incomes (M = 1,314,000 Tomans; SD = 800.89; approximately $35 USD at the time of data collection). Educational attainment varied, with 37.5% having completed primary school, 37.5% lower secondary school, and 25% holding a high school diploma.

#### Procedure

The study protocol received full approval from the University of Kurdistan Research Ethics Committee (ID: IR.UOK.REC.1398.046) and was conducted in accordance with the Declaration of Helsinki. It should be noted that the study was not formally registered in a clinical trial registry prior to participant enrollment, as it was designed as an early-phase, pilot evaluation using a single-case experimental design, for which registration was not standard practice at the time of study initiation.

A key ethical consideration was ensuring that the recruitment and screening process was non-coercive and respectful of participants' vulnerability. As such, no direct contact was made by the research team until a potential participant had first been approached by a trusted shelter staff member and had explicitly agreed to a meeting. Furthermore, all research personnel who had direct contact with participants (the second author and the facilitators) had completed specialized training in the ethical conduct of research with vulnerable populations, including trauma-informed principles of communication. This training was designed to foster interactions conducted with the utmost sensitivity to the participants' experiences.

Women who expressed interest were then formally screened for eligibility. Inclusion criteria were established to create a relatively homogenous sample, appropriate for this early-phase evaluation. Participants were required to be: (a) between 18 and 50 years of age, a range selected to represent the primary child-rearing years; (b) have experienced IPV within the preceding two years, with the aim of including participants whose traumatic experience was relatively recent; (c) have at least one child, as the intervention is parenting-focused; (d) possess a minimum of an elementary school education, considered necessary for comprehension of the program's psychoeducational materials and self-report measures; and (e) provide written informed consent.

Exclusion criteria were established to minimize potential confounding variables and to mitigate risks to participant safety. These were: (a) a current diagnosis of a substance use disorder or psychosis, as these conditions could interfere with group processes; (b) concurrent use of certain psychotropic medications, in order to better isolate the effects of the psychosocial intervention; or (c) participation in a similar psychological intervention within the past year, to avoid potential carry-over effects. Eligibility was ascertained via self-report during a structured screening interview.

A comprehensive crisis management and safety protocol, designed to leverage the existing supportive infrastructure of the collaborating shelters, was established and reviewed with all participants during the informed consent process. This protocol included several key components: First, participants were monitored throughout the study for signs of acute psychological distress (e.g., severe emotional reactions, suicidal ideation) or disclosures of imminent danger. Second, a clear referral pathway was established with the shelters' on-site clinical staff. In the event that a participant exhibited acute distress or reported being in immediate danger, the protocol required facilitators to pause the session and immediately refer and accompany the participant to on-site clinical services for assessment and support. Third, all participants were provided with contact information for around-the-clock support, including local emergency services, a domestic violence hotline, and the lead shelter clinician. The objective of this multi-pronged protocol was to provide a clear mechanism for addressing any emergent risks swiftly and effectively, thereby prioritizing participant safety above all research objectives.

Following the completion of all screening and consent procedures, the study unfolded across three phases. The baseline phase (A) consisted of three weekly assessments of participants' PTSS and depressive symptoms. The intervention phase (B) commenced immediately following the baseline, during which the 10-week MEP was delivered. Assessments continued on a weekly basis throughout this phase. Finally, the follow-up phase involved three additional assessments conducted at one, two, and three months post-intervention to evaluate the maintenance of therapeutic gains.

#### Intervention and Cultural Adaptation: Moms’ Empowerment Program (MEP)

The intervention was the Moms’ Empowerment Program (MEP), a manualized, 10-session group psychoeducational program based on the protocol by Graham-Bermann et al. [33]. Each 90-min session was conducted weekly by two trained female facilitators. To ensure treatment fidelity, facilitators (a) received 20 h of intensive training on the MEP manual, (b) participated in weekly supervision meetings with the principal investigator to review session adherence and address challenges, and (c) utilized a session-by-session checklist to verify that all core programmatic components were delivered as intended.

A key component of this study was the systematic, multi-layered cultural adaptation of the Mom's Empowerment Program (MEP) for the Iranian context. To ensure fidelity to the program's core theoretical principles, the initial adaptation was conducted by the program's developer (a co-author). This initial version was then subjected to a rigorous review by the Iranian co-authors; drawing on their extensive clinical and research experience in Iran, they endorsed the adaptation, finding it culturally and conceptually appropriate without requiring modifications.

This multi-stage process aimed to achieve conceptual and functional equivalence, moving beyond simple linguistic translation. Following the conceptual validation of this foundational version, the next step was a formal back-translation protocol to ensure linguistic fidelity. Subsequently, an expert panel was convened, comprising three local mental health professionals (a family therapist, a clinical psychologist, and a social worker) with substantial experience working with IPV survivors. The panel's task was to scrutinize the manual for cultural relevance and identify potential areas of friction.

For instance, the 'Safety Planning' module (Session 7) required significant modification. The original protocol emphasized formal resources, such as police intervention and shelters. The panel noted that due to cultural values surrounding family privacy, many women would first seek informal support. Consequently, the module was expanded to include specific strategies for identifying and engaging trusted allies within the extended family and community, thereby creating a culturally congruent safety network. Similarly, the concept of 'empowerment' was reframed. Rather than focusing exclusively on individualistic self-assertion, discussions were guided to frame empowerment as strengthening a woman's capacity to protect and nurture her children and family, an interpretation aligned more closely with the participants' collectivistic values.

The adaptation process also involved addressing potential cultural dilemmas. A key point of discussion was how to approach topics like divorce in a manner that was empowering without being prescriptive or violating cultural norms that emphasize marital preservation. The panel agreed to adopt a psychoeducational, non-directive stance. In this role, facilitators provide comprehensive information about all available legal and social options, while the therapeutic focus remains on enhancing safety and well-being, regardless of the woman's decision about her marital status. This integration of culturally resonant elements, such as relevant proverbs and verses from Rumi that frame resilience as a spiritual virtue, was intended to enhance the program's relevance, acceptability, and ultimately, its therapeutic efficacy. A detailed outline of the final session topics is provided in Table 1.

**Table 1:** Mom’s Empowerment Program (MEP): Session Outline Based on Graham-Bermann et al. [33]

Session		Session Focus	Objectives
1	Establishing the Group		To establish the group as a safe space and a source of support, focusing on the empowerment of women surviving domestic violence
2	Mothers' Fears & Concerns; Parenting Under Stress		To explore parenting concerns and connect the tactics of domestic violence to the experience of parenting stress
3	Child Development: Typical vs. Atypical		To increase understanding of the effects of domestic violence on children's social, emotional, and cognitive development, and to differentiate between typical and atypical behaviors
4	Communicating About Domestic Violence		To help mothers develop strategies for positive communication with their children, aimed at enhancing children's coping skills and reducing parenting challenges
5	Family of Origin and Intergenerational Patterns		To support mothers in developing parenting approaches that differ from the patterns learned in their own families of origin
6	Discipline Strategies: What Works?		To identify challenges in child behavior management and to share information on effective and non-violent discipline techniques
7	Safety Planning and Community Resources		To address the stress associated with accessing social services and to provide information on available community, economic, and emotional support resources
8	Stress Management for Mothers and Children		To facilitate a group discussion on positive and negative coping strategies for parenting stress, with an emphasis on exploring the role of spirituality and faith
9	Making Time for Fun and Positive Interaction		To introduce the concept of positive reinforcement, emphasizing praise and attention to a child's strengths to reduce negative behaviors and enhance the parent–child relationship
10	Program Review and Closure		To review and process the group's accomplishments to consolidate gains. Includes saying goodbye to group members and holding a graduation ceremony for the mothers

#### Measures

A Demographic Questionnaire was administered to collect data on participants' age, marital status, education, income, and family composition.

PTSD Checklist for DSM-5 (PCL-5). The PCL-5 [34] is a 20-item self-report measure assessing the severity of PTSD symptoms corresponding to DSM-5 criteria. Items are rated on a 5-point Likert-type scale (0 = Not at all to 4 = Extremely). The PCL-5 has demonstrated excellent psychometric properties, including high internal consistency in its original validation (α = 0.97). The Iranian validation by Varmaghani et al. [35] confirmed its utility, reporting strong internal consistency (α = 0.92) and evidence of convergent validity through a significant positive correlation with a measure of depression (r = 0.78, p < 0.001). In the present study, the Cronbach's alpha for the PCL-5 total score at baseline 0.94 at baseline and 0.72 at the 3-month follow-up.

Beck Depression Inventory-Short Form (BDI-SF). The 13-item BDI-SF [36] is a widely used self-report instrument for measuring the severity of depressive symptoms [37]. Each item is rated on a 4-point scale (0–3. The BDI-SF has well-established psychometric properties, including high internal consistency in samples of women who have experienced IPV (α = 0.88 [38],). The Iranian validation study by Hamidi et al. [39] also reported excellent internal consistency (α = 0.93). In the current sample, the Cronbach's alpha for the BDI-SF at baseline was 0.91 and 0.85 at the 3-month follow-up.

#### Data analysis

Given the single-case experimental design (SCED) of the study, the primary method for data analysis was systematic visual analysis, which is considered the cornerstone for interpreting SCED data [40]. This involved plotting each participant's PCL-5 and BDI-SF scores across the baseline, intervention, and follow-up phases. The plots were systematically inspected for immediate and sustained changes in three key features: level (mean score), trend (slope), and variability following the introduction of the MEP. While quantitative analyses such as Repeated Measures ANOVA were considered, they were deemed inappropriate given the small sample size (*N* = 8) and the primary focus of SCED on individual-level change rather than group-level averages.

To supplement the visual analysis and provide a quantitative estimate of the intervention's magnitude, between-phase effect sizes were calculated. Specifically, Cohen's d was computed for each participant by comparing their mean scores during the baseline phase to their mean scores during the follow-up phase, providing a robust measure of individual treatment effect. All participants completed all scheduled assessments across all phases of the study; consequently, there were no missing data to address.

## Results

The primary analysis involved a systematic visual inspection of each participant's PTSS (PCL-5) and depression (BDI-SF) scores across all study phases. This visual analysis, which is the cornerstone of single-case experimental design evaluation [40], was supplemented with quantitative analysis, including the calculation of between-phase effect sizes (Cohen's *d*).

### Visual analysis of treatment effects

**Fig. 1:**
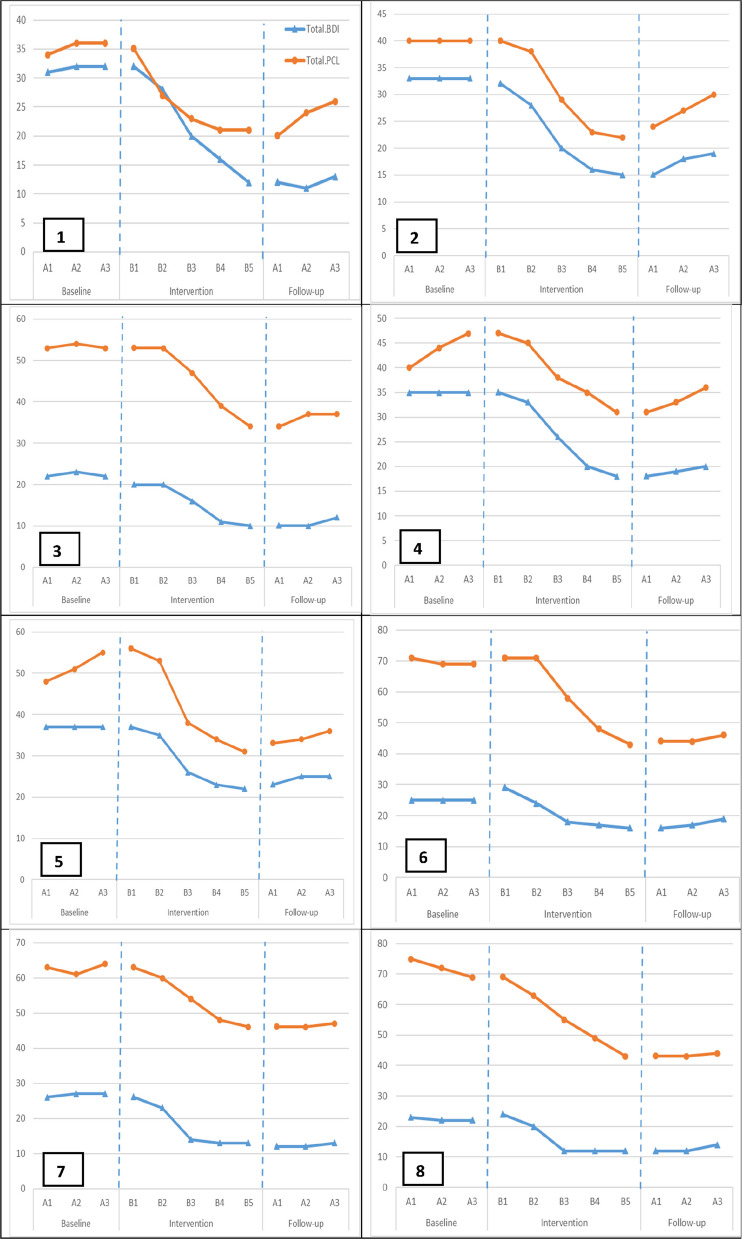
Post-Traumatic Stress Symptoms (PCL-5) and Depressive Symptoms (BDI-SF) Scores across Baseline, Intervention, and Follow-up phases for all eight participants

The plotted data for all eight participants are presented in Fig. 1. A consistent and clear therapeutic effect was observed across both dependent variables following the introduction of the Moms’ Empowerment Program (MEP).

During the baseline phase (A), participants' scores on the PCL-5 and BDI-SF were consistently elevated and demonstrated a relatively stable pattern, indicating significant and persistent PTSS and depressive symptoms.

Upon commencement of the intervention phase (B), a marked and immediate change in the data pattern was evident for all eight women. There was a clear change in level, with scores beginning a consistent downward trend. This therapeutic trajectory continued throughout the 10-week intervention, indicating a robust response to the treatment.

In the follow-up phase, conducted over the three months post-intervention, the therapeutic gains were fully maintained or, in some cases, continued to improve slightly. Participants' scores on both the PCL-5 and BDI-SF remained stable at these new, significantly lower levels, demonstrating the durability of the intervention's effects.

To quantify the magnitude of the observed therapeutic effects, descriptive statistics for each phase and between-phase effect sizes were calculated (see Table 2). The data confirm the visual analysis, showing a substantial reduction in mean scores from the baseline to the follow-up phase for both PTSS (from M = 57.04 to M = 33.71) and depression (from M = 30.17 to M = 15.63).

**Table 2:** Descriptive Statistics and Between-Phase Effect Sizes for PTSS and Depressive Symptoms (*N* = 8)

Participant	variable	Tau-U	NAP (%)	PAND (%)	RCI
1	BDI	.83	87.5	87.5	–80.29
	PCL	.92	95.8	87.5	–30.00
2	BDI	1	100	100	–66.68
	PCL	.96	95.8	87.5	–68.33
3	BDI	.00	100	100	–47.63
	PCL	.83	87.5	75	–86.67
4	BDI	.92	91.7	87.5	–34.02
	PCL	.62	79.2	75	–8.49
5	BDI	.96	95.8	87.5	–32.21
	PCL	.58	79.2	87.5	–13.97
6	BDI	.75	87.5	100	–32.66
	PCL	.58	75	75	–73.13
7	BDI	.96	95.8	87.5	–58.52
	PCL	.87	91.7	87.5	–30.87
8	BDI	.75	87.5	100	–39.46
	PCL	.96	95.8	87.5	–27.58

*M* Mean, *SD* Standard Deviation, *Tau-U* The Kendall Tau-U Coefficient, *NAP* Nonoverlap of All Pairs, *PAND* Percentage of All Nonoverlapping Data, *RCI* Reliable Change Index, *BDI* Beck Depression Inventory-Short Form, *PCLPTSD* Checklist for DSM-5

Effect sizes were calculated for each participant by comparing scores during the three-week baseline phase to mean scoresduring the three-month follow-up phase

To assess the clinical significance of these changes at the individual level, Cohen's *d* was calculated for each participant, comparing their mean baseline scores to their mean follow-up scores. As shown in Table 2, the effect sizes were consistently large across all participants for both outcomes. For PTSS, Cohen's *d* ranged from 1.83 to 1.83. For depressive symptoms, Cohen's *d* ranged from 1.64 to 1.82. According to conventional standards [41], these effect sizes are indicative of a powerful and highly significant treatment effect.

To explore the clinical significance of these changes, a cut-off score of ≥ 33 on the PCL-5 was utilized. This threshold is consistent with psychometric studies and is commonly recommended as a robust indicator of clinically significant post-traumatic stress symptoms (PTSS) requiring clinical attention [34]. At baseline, all eight participants (100%) scored above this threshold. By the final follow-up assessment, two of the eight participants (25%) scored below this clinical cut-off. Importantly, this finding should be viewed in the context of the substantial and large-magnitude reductions in PTSS scores observed for all participants, as evidenced by the consistently large Cohen's d effect sizes reported in Table 2.

At the group level, the intervention demonstrated substantial and clinically meaningful effects, particularly in reducing depressive symptoms and trauma-related distress. Using the repeated-measures adjusted Cohen’s *d*, the effect size for depressive symptoms (BDI) was large (*d repeated-measures* = 1.52), indicating a robust change over time. The effect for PTSS (PCL), while more moderate (*d repeated-measures* = 0.44), remained statistically and clinically relevant. These findings were further supported by Hedges’ *g* (1.37 for BDI; 0.39 for PCL), which accounts for sample size correction and is commonly preferred in studies with small sample sizes.

## Discussion

This study provides compelling evidence for the efficacy of a culturally adapted Moms’ Empowerment Program (MEP) in mitigating the severe psychological sequelae of intimate partner violence (IPV) among a sample of Iranian mothers. The findings were not merely statistically significant; the visual analysis revealed an immediate, consistent, and durable therapeutic effect for all eight participants, a conclusion strongly corroborated by large and robust effect sizes (Cohen's d = 1.64–1.83). This discussion seeks to interpret the mechanisms driving this profound change, situate the findings within the broader scientific literature, and consider their implications for both theory and practice.

The substantial improvements observed in this study can be attributed to a powerful synergy between two distinct yet intertwined sets of mechanisms, a distinction central to psychotherapy research: (a) intervention-specific factors stemming from the MEP's structured, skills-based curriculum, and (b) common or non-specific therapeutic factors inherent to the supportive group modality itself [42]. The ensuing discussion will first unpack the specific components of the MEP curriculum that likely drove the reduction in PTSS and depressive symptoms, before examining the foundational role of the group context in facilitating this change.

The observed reductions in trauma-related symptoms (as measured by the PCL-5) and depressive symptoms (as indicated by the BDI-SF) reflect clinically meaningful changes. While these changes do not imply the elimination of PTSS or depression diagnoses per se, they signify substantial alleviation in symptom severity, functional burden, and emotional distress. Such results are particularly promising given the short duration of the intervention and the severity of participants' baseline distress. These symptom improvements align with evidence from prior studies on MEP in Western contexts [26, 43, 44], but the consistency and strength of change across all participants in this cultural context are especially noteworthy.

The powerful therapeutic effects of the MEP can be understood as a systematic process of psychological re-empowerment that directly counters the cognitive and behavioral paralysis described in the theory of learned helplessness [45], which has been applied to the experience of battered women by Walker [28].

The program appears to function as a powerful engine for rebuilding a sense of personal agency and self-efficacy—the belief in one's capacity to effect change. This is achieved not through abstract discussion, but through structured, experiential learning. For example, when mothers in Session 7 develop and rehearse a concrete safety plan, they move from a passive state of fear to a proactive stance of strategic control. Each successfully navigated challenge, whether it be setting a boundary or employing a new parenting technique, serves as a powerful mastery experience that provides tangible evidence against the internalized narrative of helplessness.

It is also important to recognize that the impact of IPV on parenting is not uniform; research has identified distinct parenting profiles among mothers who have experienced violence, ranging from those who maintain positive parenting to those who exhibit more significant difficulties [30]. Furthermore, the program's explicit focus on parenting (e.g., Session 5, "Family of Origin") provides a deeply meaningful and accessible domain for mothers to exercise this newfound agency. By helping them develop parenting approaches that differ from the patterns of violence they may have learned, the MEP empowers them to actively break an intergenerational cycle. This fosters a sense of purpose and competence that can generalize to other areas of their lives, creating a virtuous cycle of empowerment. From a clinical perspective, improvements in parenting self-efficacy may indirectly reinforce emotional stability and reduce depressive symptomatology, as suggested in other maternal mental health research. This empowerment in the parenting domain is not merely theoretical,for instance, participation in the MEP has been shown to significantly reduce mothers' use of corporal punishment, directly interrupting a cycle of violence [27].

In addition, PEM specifically addresses stress management in Session 8. This session first provides a space for mothers to name specific stressors in their own lives as well as in the lives of their children, which in itself potentially reduces psychological burden through naming and validating the experience. It then also provides a range of concrete, healthy strategies for managing distress—behavioral activities through relaxation strategies and through empowering purposeful actions such as returning to normal life. Notably, participants are encouraged to move forward with strategies that have already been tried, with constructive feedback in a nonjudgmental environment. The program provides mothers with skills for managing distress in the present moment that directly address the symptoms of hyperarousal and emotional regulation issues that are associated with the symptoms of posttraumatic stress.

The high retention rate (zero attrition) and consistent engagement of the participants also speak to the feasibility and high acceptability of this culturally adapted model. In a context where formal mental health services can be stigmatized or perceived as culturally incongruent [8], a group-based, skills-focused program centered on a valued social role—motherhood—appeared to be a highly accessible and non-threatening entry point for support. The inclusion of culturally resonant elements likely enhanced this acceptability, a finding consistent with the broader literature on cultural adaptation, which posits that such modifications increase engagement and relevance [46]. This suggests that the MEP's framework is not only effective but also a viable and well-received model for delivering mental health support to this vulnerable and underserved population.

The important component is that these intervention mechanisms worked in a supportive group setting, which provided essential non-specific therapeutic factors for recovery. The concept of universality, realizing that one is not alone in their suffering [47], can significantly reduce the shame and isolation that often come with trauma. In this study, participants consistently expressed feelings of "being seen" and "being understood," which helped shift their emotional distress from an individual flaw to a collective human experience. This supportive environment was certainly a key component. However, the synergy of this safe space with the organized and skill-based MEP is likely what explains the significant and lasting effects observed. The group makes change safe, and the curriculum provides participants the tools they need to do it.

A signal contribution of this study is its demonstration of successful cultural adaptation. The integration of inspirational readings from the Quran and the poetry of Rumi aligns the intervention with the participants' sociocultural and spiritual worldview. This is not a superficial garnish but a deep structural enhancement that likely increases the program's acceptability, relevance, and power [46]. These elements served not only to frame therapeutic content in familiar language, but also to invoke culturally resonant narratives of endurance, moral strength, and hope. By aligning therapeutic goals with spiritual identity, the program may have activated indigenous coping strategies and resilience pathways not typically accessed in Western protocols.

The results of this study make a dual contribution to the existing literature. First, they strongly corroborate the findings of previous research in Western contexts, which have consistently demonstrated the MEP’s efficacy in reducing maternal PTSS and depressive symptoms [26, 44, 48]. Our study affirms that the core principles of the MEP are robust. Second, and more significantly, this study extends the literature by providing the first, to our knowledge, evaluation of the MEP in a Middle Eastern, specifically Iranian, population. This is a critical extension, as interventions for IPV survivors are predominantly developed and tested in high-income, Western nations, leaving a substantial gap in evidence for regions like Iran, where the burden of IPV is particularly severe.

### Limitations and future directions

While the findings of this study are encouraging, several methodological limitations warrant careful consideration. The use of a Single-Case Experimental Design (SCED), although well-suited for this early-phase evaluation of individual change, does not permit strong causal inferences or full control over non-specific therapeutic factors, such as the passage of time or attention effects. Although SCEDs utilize participants' repeated baseline assessments as an internal control, the lack of a separate, independent comparison group constrains the ability to attribute observed changes exclusively to the MEP intervention. Furthermore, the small sample size (*N* = 8), drawn from a specific geographic and cultural setting, necessarily limits the generalizability of the findings to the broader population of Iranian mothers who have experienced IPV.

Conceptually, it is important to acknowledge that the MEP is a survivor-focused intervention and does not directly engage the abusive partner or address the perpetration of violence. The program's primary aim is to mitigate the psychological sequelae of IPV and enhance the mother's coping and parenting capacities, thereby empowering her to create a safer and more stable environment for herself and her children. While this is a crucial component of recovery, such interventions cannot substitute for broader systemic responses that address the source of the violence, including legal protections and perpetrator intervention programs. Future research could productively examine the synergistic effects of integrating survivor-focused programs like the MEP with these more comprehensive, systemic interventions.

Methodologically, the exclusive reliance on quantitative self-report measures provides a clear account of score changes but may not fully capture the qualitative nature of participants' improvement. The inclusion of a qualitative component, such as post-intervention interviews, could have offered rich insights into participants' perceptions of change, the programmatic elements they found most beneficial, and their experience of skill acquisition. Future research would benefit from a mixed-methods design to triangulate these quantitative findings and deepen the understanding of the MEP's mechanisms of impact.

Finally, the analysis of clinical significance offers a crucial insight into the precise role and scope of this psychoeducational intervention. While the program catalyzed substantial reductions in symptom severity for all participants, full clinical remission—defined as falling below the clinical threshold for PTSS—was achieved by a quarter of the sample. This finding should not be viewed as a shortcoming but rather as a clear reflection of the intervention's intended function. As a structured, skills-based program, the MEP is designed to be a potent foundational intervention, not a comprehensive trauma-processing psychotherapy. Its primary objective aligns with the first phase of phase-oriented trauma therapy: establishing safety and stabilization [49, 50]. The results support the view that the MEP is effective in this role. However, the data also suggest that for a significant portion of survivors, processing the deeper wounds of complex trauma may necessitate a subsequent, more intensive therapeutic phase [51]. This strongly argues for future research to explore adaptive, stepped-care models, where a program like the MEP serves as the critical first tier, preparing and stabilizing survivors for deeper therapeutic work if and when it is needed [52].

### Implications for practice

These findings offer strong preliminary support for the use of the Moms’ Empowerment Program (MEP) as a structured, time-limited, and effective intervention for reducing trauma- and depression-related symptoms in mothers affected by intimate partner violence. For practitioners working in community-based settings—such as women’s shelters, social service agencies, and public mental health clinics—this 10-session group model represents a practical, evidence-informed alternative to unstructured support groups or longer-term individual therapies that may be less feasible in resource-constrained environments. This aligns with broader calls within the field to move toward structured, evidence-based programming, ensuring that interventions are not only supportive but also demonstrably effective [53].

Moreover, this study underscores the critical importance of cultural adaptation in clinical practice. The successful incorporation of locally meaningful materials—such as Quranic verses and classical Persian poetry—not only enhanced the cultural relevance of the program, but may also have deepened its emotional resonance and therapeutic impact. These findings highlight that effective care is not simply about fidelity to Western evidence-based models, but also about the thoughtful integration of clients’ cultural worldviews, values, and traditions. When clinicians actively engage in such a culturally attuned process, they can foster greater engagement, strengthen therapeutic alliance, and ultimately promote more sustainable psychological change.

## Conclusion

In conclusion, this study offers compelling preliminary evidence of meaningful therapeutic change. For the eight women who participated, engagement in a culturally adapted empowerment program was associated with a substantial and sustained reduction in trauma- and depression-related symptoms. These outcomes appear to have been driven by a synergy between structured, skills-based interventions that restored a sense of agency and a deep cultural attunement that honored participants’ values, narratives, and sources of strength.

While further validation through larger-scale trials is essential, the present findings signal both promise and direction. They point toward a model of care that is not only effective but also contextually respectful—one that recognizes empowerment as both a psychological and cultural process. For clinicians and policymakers alike, these results underscore a clear imperative: supporting mothers in reclaiming their agency is a direct and vital path toward repairing the harms of violence and restoring the foundational stability of the family.

## Data Availability

The datasets generated and analyzed during the current study are not publicly available due to ethical constraints but may be made available from the corresponding author on reasonable request.
